# Supplementary material for: The interactome of intact mitochondria by cross-linking mass spectrometry provides evidence for coexisting respiratory supercomplexes
Source: Mol Cell Proteomics. 2017 Dec 8;17(2):216–32. doi: 10.1074/mcp.RA117.000470 (PMC5795388; doi:10.1074/mcp.RA117.000470)
Supplement: Supplemental Data [file supp_RA117.000470_133922_0_supp_23978_fzffwf.zip › spectra_annotation/mito_DR_spectra_annotation/140-1-6-1-6-1.pdf]

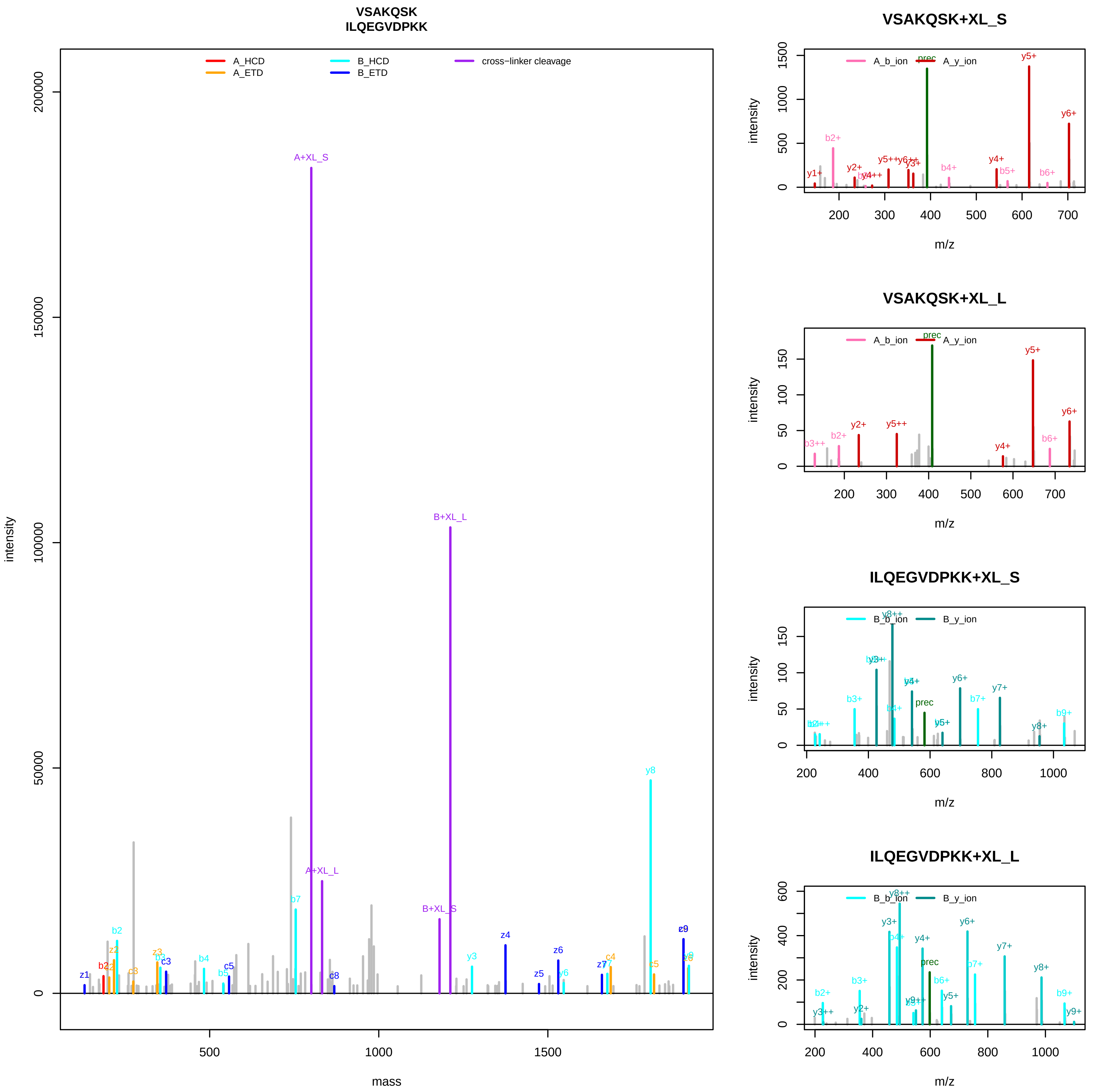
VSAKQSK
ILQEGVDPKK
A_HCD
A_ETD
B_HCD
B_ETD
cross−linker cleavage
intensity
mass
0
50000
100000
150000
200000
500
1000
1500
A+XL_S
B+XL_L
A+XL_L
B+XL_S
y8
b7
z9
c9
z4
z6
y3
z5
y6
z7
c4
c5
c8
c5
b5
b4
c3
z3
b3
c3
b2
z2
z1
VSAKQSK+XL_S
A_b_ion
A_y_ion
prec
y5+
y6+
b2+
y2+
y1+
y5++
y6++
y3+
y4++
b4+
y4+
b5+
b6+
m/z
VSAKQSK+XL_L
b3++
b2+
y2+
y5++
y4+
b6+
ILQEGVDPKK+XL_S
B_b_ion
B_y_ion
y8++
y3+
b3+
y4+
b4+
y6+
y7+
b7+
b9+
y8+
y5+
ILQEGVDPKK+XL_L
y3+
b4+
y4+
y6+
y7+
b7+
b6+
y5+
y8+
b9+
b2+
b3+
y2+
y3++
y9++
y9+
